# Transcultural adaptation and validation of a Korea version of Pedi-IKDC questionnaire

**DOI:** 10.1186/s13690-023-01236-7

**Published:** 2024-01-18

**Authors:** Woo Sub Kim, Seung Hyun Won, Seo Ho Moon, Min Joon Oh, Moon Seok Park, Ki Hyuk Sung

**Affiliations:** 1 Department of Orthopaedic Surgery, Seoul National University College of Medicine, Seoul National University Bundang Hospital, 82 Gumi-ro 173 Beon-gil, Bundang-Gu, 13620 Sungnam, Gyeonggi Korea; 2 Division of Statistics, Medical Research Collaborating Center, Seoul National University Bundang Hospital, Gyeonggi, Korea; 3 Department of Pathology, Duke University School of Medicine, Durham, NC USA

**Keywords:** Pedi-IKDC, Translation, Transcultural adaptation, Validation

## Abstract

**Background:**

This study aimed to translate and transculturally adapt the English version of the Pedi-IKDC questionnaire into Korean and evaluate the psychometric properties of the Korean Pedi-IKDC questionnaire in terms of internal consistency, feasibility (floor and ceiling effect), construct validity, test-retest reliability, and factor analysis.

**Methods:**

The original English version of the Pedi-IKDC questionnaire was translated and transculturally adapted into Korean according to established guidelines. A total of 239 patients aged 7–18 years who visited the hospital because of knee pain or discomfort were considered eligible for the study. These patients completed the Korean version of the Pedi-IKDC and Pediatric Quality of Life questionnaires (PedsQL). The correlation between the PedsQL and Pedi-IKDC questionnaires was assessed to confirm the validity of the questionnaire. To verify the validity of the Korean Pedi-IKDC questionnaire, internal consistency, feasibility, test-retest reliability, and construct validity were evaluated, and a factor analysis was performed.

**Results:**

Internal consistency was found to be satisfactory in all subscales (Cronbach’s alpha ≥ 0.7). The test-retest reliability was satisfactorily high for all subscales (Intraclass correlation coefficient: 0.81–0.84). A high correlation was observed between the total Pedi-IKDC score and the score on the physical-health subscale of child version of the PedsQL (Correlation coefficients: 0.720). There were no floor effects in all subscales, but ceiling effects were observed in four questions. Additionally, factor analysis suggested that the questionnaire could be divided into two subscales.

**Conclusion:**

The Korean version of the Pedi-IKDC questionnaire was successfully translated and transculturally adapted according to the established guidelines. The Korean Pedi-IKDC questionnaire has been proven reliable and valid.

**Table:** 

Text box 1. Contributions to the literature
• Children’s health is of great importance to public health and the national healthcare system.
• The level of discomfort in their daily lives can be assessed through a questionnaire, and such assessments can be efficiently utilized in public health and healthcare systems.
• Even if the original questionnaire is considered a reliable one, without appropriate transcultural adaptation and validation, the psychometric properties of the questionnaire may change, making evaluation essential.
• The Korean version of the Pedi-IKDC questionnaire can be used for effective evaluation, contributing to research on the public healthcare system related to children’s knee pain.

## Background

Owing to increased physical activity, many children and adolescents may experience knee pain with or without injury. Experts treat patients with medications, orthoses, and surgery. After treatment, attempts are usually made to assess the treatment outcomes clinically. However, the results of these assessments do not reflect social functioning and patients’ daily quality of life [1]. For this reason, various questionnaires have been studied to confirm subjective discomfort, social functioning, and patient’s daily quality of life [2]. Questionnaires specifically for children to confirm their subjective discomfort, social functioning, and daily quality of life are very important for several reasons [3]. First, children’s physical abilities affect their relationships with friends and significantly affect their physical and mental development [4]. Second, there are differences between the daily and sporting activities of children and adults [5]. Third, asking questions regarding activity levels according to age is necessary [6]. Fourth, parental help may be required to complete the questionnaire.

The International Knee Documentation Committee (IKDC) questionnaire is widely used as a questionnaire focusing on the knee, the Pedi-IKDC questionnaire, which is modified for children, has been developed [2]. The psychometric properties of the Pedi-IKDC questionnaire have already been evaluated, and acceptable results have been obtained for reliability, validity, and responsiveness [7]. One previous study showed that the Pedi IKDC questionnaire had better psychometric properties than the Knee Injury and Osteoarthritis Outcome Score for Children and should be used in children with knee disorders [8].

As appropriate questionnaires and their proper use have become more important, various questionnaires are being translated into various languages. However, the meaning and results of a questionnaire can change due to errors in the translation process and misunderstandings caused by transcultural differences. Therefore, standards for translating questionnaires into other languages and transcultural adaptation to the country have been established. Additionally, several methods exist to test the psychometric properties of the translated questionnaire [9].

Therefore, this study aimed to translate and transculturally adapt the English version of the Pedi-IKDC questionnaire into Korean and evaluate the psychometric properties of the Korean Pedi-IKDC questionnaire in terms of internal consistency, feasibility (floor and ceiling effect), construct validity, test-retest reliability, and factor analysis.

## Methods

### Ethical statements

This study was approved by the Institutional Review Board of our hospital, and informed consent was obtained from the parents or guardians of all patients.

### Translation and transcultural adaptation

The original English version of the Pedi-IKDC questionnaire was translated and transculturally adapted into Korean according to established guidelines [9, 10].

1. Forward translation.

The English version of the Pedi-IKDC questionnaire was translated into Korean by two translators who were native Korean speakers fluent in English. Each translator worked independently; one was an orthopedic surgeon, and the other was a non-medical translator.

2. Reconciliation.

A consultation team comprising six members held a meeting. The five members included three orthopedic surgeons, a regular nurse, and a research assistant, who reconciled the two original Korean versions of the Pedi-IKDC questionnaire into a single Korean version.

3. Back translation.

The reconciled Korean version of the Pedi-IKDC questionnaire was back-translated into English by two bilingual Korean-American native English speakers who worked independently. One translator was a professional medical translator, while the other was a non-medical translator; both translators were blinded to the original English version of the Pedi-IKDC questionnaire.

4. Harmonization.

A consultation team reviewed the original English and back-translated versions of the Pedi-IKDC questionnaire. The consultation team had seven members: three orthopedic surgeons, one registered nurse, one research assistant specializing in orthopedic scoring systems, a medical professional, and a non-medical professional. The consultation team compared each item of the back-translated and original versions of the Pedi-IKDC questionnaire in terms of semantic, idiomatic, experiential, and conceptual equivalence. Members evaluated the degree of equivalence for each item as equivalent, partially equivalent, or different. Upon completing the analysis of all items, the final Korean version of the Pedi-IKDC was created after discussing and revising the items evaluated as partially equivalent or different.

5. Cognitive debriefing.

The final version of the Korean Pedi-IKDC questionnaire was preliminarily administered to 15 patients with knee pain who visited an outpatient clinic. A research assistant interviewed patients and their parents to investigate whether there were any problems in understanding and answering the questions for each item in the Korean Pedi-IKDC questionnaire. Both patients and their parents fully understood the questions for each item and had no difficulty answering the questionnaire.

6. Proofreading.

All proofreading errors were corrected before the final Korean version of the Pedi-IKDC questionnaire was produced.

### Participants and testing the psychometric properties of the Korean Version of the Pedi-IKDC questionnaire

From August 2020 to April 2022, a study was conducted on 239 patients aged between 7 and 18 years who visited the hospital for knee pain or discomfort and whose caregivers provided informed consent. The patients completed the Pedi-IKDC questionnaire with the help of their caregivers. The Pediatric Quality of Life (PedsQL) questionnaire was also completed simultaneously, and the correlation between both questionnaires was analyzed for each subscale to confirm construct validity.

The Pedi-IKDC questionnaire is a knee-related questionnaire consisting of 17 Likert-based questions and 4 visual analog scale-based questions consisting of 3 dimensions: symptoms (9 items), sporting activities (2 items), and function (2 items), for a total of 13 items [2]. Additionally, it includes a scoring form that measures the scores based on a questionnaire completed by the patient. The scores range from 0 to 100 on a functional scale; the higher the score, the lesser the pain, the fewer the symptoms, and the higher the level of physical function. The PedsQL questionnaire uses a 5-point (0–4) Likert scale to assess the 4 dimensions of physical functioning (8 items), emotional functioning (5 items), social functioning (5 items), and school functioning (5 items) for a total of 23 items [11]. Higher scores indicate better health-related quality of life. The PedsQL questionnaire has two versions: one for children and one for caregivers because patients and caregivers are required to complete separate questionnaires. For 23 patients, test-retest reliabilities were assessed with 4-week intervals to prevent memory contamination. These patients underwent further evaluation, such as laboratory exam, CT, or MRI without taking medications [12]. All steps were performed under the supervision of a research assistant. The questionnaire’s psychometric properties were evaluated as recommended in the international CONsensus-based Standards for the selection of health status Measurement INstruments (COSMIN) guideline [13, 14]. The feasibility, internal consistency, construct validity, and test-retest reliability, were evaluated to confirm the psychometric properties. Factor analysis was also performed.

We hypothesized that the physical subscale of the PedsQL questionnaire completed by patients and their caregivers would significantly correlate with the Pedi-IKDC questionnaire, which assesses the degree of physical activity and daily quality of life possible with knee pain.

### Statistical analysis

Cronbach’s alpha (α) was used to examine internal consistency, and values ≥ 0.7 were considered satisfactory [15]. The questionnaire’s reliability was assessed by using the test-retest reliability method to measure the intraclass correlation coefficient (ICC; two-way random model, single measure); if the ICC was ≥ 0.7, it was considered a reliable questionnaire [12, 16]. The correlation between the Pedi-IKDC and PedsQL subscales was analyzed to confirm the construct validity of the Pedi-IKDC questionnaire. Pearson’s correlation coefficients were used for the correlation coefficient, and the correlation was considered as low (*r* < 0.3), moderate (0.3 ≤ *r* < 0.7), or high (*r* ≥ 0.7) [17]. Kaiser-Meyer-Olkin (KMO) measure and Bartlett’s sphericity tests were performed to confirm whether the samples were appropriate for factor analysis. A raw score was identified and used for varimax rotation, which is an orthogonal rotation, to interpret the factor loadings. Factor loadings > 0.5 were considered indicative of item loading [18]. Since floor and ceiling effects can affect reliability, validity, and responsiveness, a floor or ceiling effect was considered to exist if the highest and lowest scores were observed for > 30% of the total respondents [19]. R Version 4.0.1 (R Foundation for Statistical Computing) and R Studio Version 1.3.959 (PBC) were used for statistical analyses. All statistics were two-tailed, and *P*-values < 0.05 were considered statistically significant.

## Results

### Translation and transcultural adaptation

During the translation and transcultural adaptation process, for questions 5 and 6, the word “puffy” was considered difficult to translate into Korean and difficult to understand because it is a word that children do not normally use. Therefore, “puffy” (or swelling) was changed to “swelling.” During the harmonization process, one item (question 10: “What is the most you can do on your injured knee most of the time?”) was considered acceptable with modifications. The two back translations of the sentence were “Recently, which of the following is the most you could do with the injured knee?” and "What are activities that you can normally do with your injured knee?” After discussion, the phrase was revised to “Recently, which of the following is the most you could do with the injured knee?” in the final Korean version of the Pedi-IKDC questionnaire.

### Psychometric properties of the Korean Pedi-IKDC questionnaire

Of the 239 patients enrolled in this study, 156 were male, and 83 were female. The average age was 13.4 ± 2.8 years. Patients’ diagnoses included anterior cruciate ligament injury, posterior cruciate ligament injury, tumor, infection and inflammation, fracture and contusion, cartilage lesion, patella dislocation, and meniscus injury (Table 1).

**Table 1:** Summary of data

Total no. of patients.	239
Mean age (SD)	13.4 (2.8)
Gender (M / F)	156 / 83
Diagnosis (No. of patients.)	
ACL injury	40(16.7%)
Tumor	18(7.5%)
Infection & Inflammation	13(5.4%)
Fracture & Contusion	18(7.5%)
Cartilage lesion	28(11.7)
Patella dislocation	41(17.2%)
Meniscus injury	52(21.8%)
PCL injury	5(2.1%)
Others (chondromalacia, tendinitis, Osgood-schlatter disease, Bipartite patella)	24(10.0%)
Pedi-IDKC subscale scores	Mean (SD)
Symptom Sports activity	25.7 (9.1) 23.0 (10.8)
Fuctional score before pain	9.3 (1.7)
Functional score after pain	5.5 (2.9)
Total score	54.2 (20.9)

SD, standard deviation

The internal consistency (Cronbach’s α) of the sports activity-related subscale was highest at 0.941, followed by that of the symptom-related subscale items at 0.807. The total score, excluding the functional subscale, was 0.930, and all subscales showed satisfactory results. The test-retest reliability was satisfactorily high for all subscales (ICC: 0.81–0.84) (Table 2).

**Table 2:** Internal consistency and test-retest reliability of the Korean version Pedi-IKDC

	Internal Consistency (Cronbach’s α)	Test-retest reliability	
		ICC	95% CI
Symptom	0.807	0.81	0.77–0.84
Sports activity	0.941	0.84	0.80–0.87
Total score	0.930	0.82	0.79–0.84

ICC, intraclass correlation coefficient; CI, confidence interval

A high correlation was observed between the total Pedi-IKDC score and the score on the physical-health subscale of child version of the PedsQL. There was a low to moderate correlation between each subscale of the Pedi-IKDC and PedsQL questionnaires (Table 3).

**Table 3:** Correlation between the Pedi-IKDC and PedsQL4.0 subscales

Pedi-IKDC		Symptom	Sports activity	Function	Total score
**Child version of PedsQL 4.0**	Physical- Health	0.673	0.667	0.584	0.720
	Emotional Function	0.267	0.321	0.246	0.317
	Social Function	0.221	0.257	0.292	0.270
	School Function	0.324	0.318	0.328	0.351
	Total score	0.494	0.518	0.470	0.549
**Caregivers version of PedsQL 4.0**	Physical- Health	0.588	0.585	0.543	0.634
	Emotional Function	0.306	0.287	0.314	0.326
	Social Function	0.199	0.242	0.257	0.247
	School Function	0.312	0.300	0.320	0.336
	Total score	0.464	0.464	0.464	0.507

The values are Pearson’s correlation coefficient values.

All values have a *P*-values < 0.05

Factor analysis suggested that the questionnaire be divided into two subscales, excluding functional subscales, similar to the original Pedi-IKDC questionnaire (Table 4).

To confirm feasibility, no floor effect was observed, and ceiling effects were confirmed in questions 5, 11b, 11e, and 11f for 107 (44.8%), 72 (30.1%), 152 (63.6%), and 130 (54.4%) patients, respectively.

**Table 4:** Factor analysis of Pedi-IKDC questionnaire

Items	Factor loading	
	Component 1	Component2
Symptom 1	0.3128	0.8537
Symptom 2	0.5777	0.2939
Symptom 3	0.3902	0.3986
Symptom 4	0.6015	0.3102
Symptom 5	0.4291	0.2626
Symptom 6	0.2866	0.7925
Symptom 7	0.1394	0.0743
Symptom 8	0.2070	0.2293
Symptom 9	0.2512	0.8947
Sports activity 10	0.4699	0.4834
Sports activity 11a	0.8454	0.2659
Sports activity 11b	0.8123	0.3110
Sports activity 11c	0.7035	0.2581
Sports activity 11d	0.7266	0.2925
Sports activity 11e	0.5934	0.1622
Sports activity 11f	0.6767	0.2361
Sports activity 11 g	0.7804	0.4383
Sports activity 11 h	0.7753	0.3876
Sports activity 11i	0.7319	0.4619

## Discussion

This study aimed to translate and transculturally adapt the English version of the Pedi-IKDC questionnaire into Korean and evaluate its psychometric properties. The translation and transcultural adaptation processes were conducted according to established guidelines, including forward and back translations, reconciliation, harmonization, cognitive debriefing, and proofreading. This study found that the Korean Pedi-IKDC questionnaire had satisfactory internal consistency, construct validity, and test-retest reliability. The factor analysis revealed that the questionnaire should be divided into two subscales in the same manner as in the original Pedi-IKDC questionnaire.

Psychometric property testing has been conducted on the Pedi-IKDC questionnaire in various languages, including English (Original) [2, 7], Danish [20], Dutch [8], Italian [21], Portuguese [22], Spanish [23], and Moroccan [6]. In all of these studies, Cronbach’s α was found to be 0.91, 0.9, 0.92, and 0.94 for English [2, 7], Danish [20], Italian [21], and Portuguese [22], respectively, demonstrating similar levels of internal consistency as observed in the present study. Additionally, this study showed excellent test-retest reliability (ICC: =0.82), consistent with other studies reporting satisfactory reliability values > 0.8 [22].

The total scores of the Pedi-IKDC and the physical health subscale of the PedsQL questionnaires for child version demonstrated a high correlation (*r* = 0.720). A low-to-moderate correlation was observed between each subscale of Pedi-IKDC and PedsQL questionnaires. The comparison of each subscale of the Pedi-IKDC with the physical health subscale of the PedsQL for both child and caregivers versions demonstrated a moderate correlation (r: 0.543 to 0.673). Similar results were obtained for the English and Portuguese versions. The English version of the Pedi-IKDC questionnaire was compared to the Child Health Questionnaire. It demonstrated the highest correlation with the physical functioning subscale, followed by the body pain and physical limitation subscales [7]. Furthermore, the Portuguese version of the Pedi-IKDC was compared with the EuroQol-5 Dimension Youth (EQ-5D-Y) and Childhood Health Assessment Questionnaire (CHAQ), which demonstrated a high correlation with the mobility, pain, usual activity, and walking subscales of the EQ-5D-Y and a high correlation with the activities (run and play) subscale of the CHAQ. However, low correlations were observed in the self-care and anxiety-related subscales of the EQ-5D-Y questionnaire [22]. While this study showed that Pedi-IKDC questionnaire had some association with the patient’s emotions, school, and social functions, these associations appeared to be less strongly related than with physical health of the PedsQL. Despite the use of different questionnaires to compare the English and Portuguese versions of the Pedi-IKDC questionnaire, the highly-correlated subscales were the same: patient physical activity and pain [7, 22]. The Pedi-IKDC questionnaire contains several questions that address physical function and pain. The high correlation observed with similar questionnaires in English, Portuguese, and Korean can be attributed to this fact, indicating the appropriateness of the translation and transcultural adaptation processes.

In the factor analysis, component one corresponded to the symptom subscale, and component two corresponded to the sports subscale. This result was similar to that of the original Pedi-IKDC questionnaire, which divided the questionnaire into symptom and sports subscales. However, owing to the small number of questions (only two) in the functional subscale and the conflicting nature of these questions, the functional subscale was excluded from the factor analysis.

There was no floor effect in all questions, and ceiling effects were confirmed in questions 5, 11b, 11e, and 11f which is similar questions and values to those of the English and Italian versions [7, 21]. Most causes of knee pain in children and adolescents are thought to have extra-articular origin [24]. For this reason, the ceiling effect observed in this study can be deduced to have occurred because the patients did not experience pain severe enough to prevent them from performing the activities mentioned in the 9 items of question 11 [25].

This study had several strengths. Our study recruited a large sample size of 239 participants and evaluated the validity of each subscale in terms of test-retest reliability, internal consistency, and construct validity. In addition, a factor analysis was conducted to confirm the two components, as in the original questionnaire.

However, this study had some limitations. First, participants were recruited from a single hospital in Korea, which may limit the generalizability of the study’s findings to other settings or populations. For the purpose of generalizability, conducting a multicenter study is necessary to target a diverse population of children and adolescents who complain of knee pain and discomfort. Second, the responsiveness to the questionnaire was not investigated. Responsiveness is considered as a measure of longitudinal validity, and a questionnaire with high responsiveness becomes an effective and reliable measurement tool for a patient’s condition [26]. To enhance the reliability and validity of the questionnaire, further studies related to responsiveness are necessary. Third, we did not conduct a prior sample size estimation considering type 1 error and statistical power, because this study did not aim to test a specific hypothesis. In our study, with 239 participants and a two-sided confidence level of 0.95, the width of the confidence interval for the Pearson correlation coefficient, which ranged from 0.2 to 0.8, was calculated to be between 0.24 and 0.09. However, we believe that the inclusion of 239 patients is sufficient to assess the clinical validity of the questionnaire, especially if compared to the sample sizes of other studies. Fourth, the authors were aware of the importance of the confirmatory factor analysis (CFA) results. However, we conducted factor analysis based on the exploratory factor analysis (EFA) results because our sample size was insufficient for the robust CFA results. Further research with a larger sample will provide a more conclusive validation of the EFA results through CFA [27].

## Conclusion

In this study, the Korean Pedi-IKDC questionnaire was successfully translated and transculturally adapted according to the established guidelines. The Korean Pedi-IKDC questionnaire has proven to be reliable and valid and a useful tool for measuring social functioning and daily quality of life in pediatric patients who complain of knee pain and discomfort.

## Data Availability

The data set supporting the conclusion of this article is available on request to the corresponding author.

## Abbreviations

IKDC: The International Knee Documentation Committee
PedsQL: The Pediatric Quality of Life
ICC: intraclass correlation coefficient
